# Kategoriale Dissonanzen. Russlands regressiver Weg in den Krieg und die Historische Soziologie imperialistischer Außenpolitiken

**DOI:** 10.1007/s42597-023-00093-z

**Published:** 2023-03-13

**Authors:** Sebastian Hoppe

**Affiliations:** Osteuropa-Institut, Freie Universität Berlin, Garystraße 55, 14195 Berlin, Deutschland

**Keywords:** Russland, Imperialismus, Souveränismus, Außenpolitik, Regression, Russia, Imperialism, Sovereigntism, Foreign policy, Regression

## Abstract

Russlands Angriff auf die Ukraine im Februar 2022 legt die Diagnose eines imperialistischen Eroberungskrieges nahe. Gleichzeitig fristen imperialismus*theoretische* Zugänge zur russländischen Außenpolitik ein widersprüchliches Nischendasein. So führen die kritischen Sozialwissenschaften Imperialismus maßgeblich auf kapitalistische Entwicklungsdynamiken und die Hegemoniebestrebungen der USA im 20. und 21. Jahrhundert zurück. Politologische und soziologische Ansätze hingegen fokussieren auf die ideologische Dimension des Imperialismus oder normalisieren imperialistische Außenpolitiken in transhistorischen Imperien-Typologien. Aufbauend auf einer Kritik bestehender Deutungsangebote unternimmt der Beitrag einen Bestimmungsversuch des russländischen Imperialismus seit den späten 2000er Jahren. Dabei wird das Argument entwickelt, dass sich in Russland vor dem Hintergrund einer Doppelkrise der Rentenökonomie und der politischen Legitimation seit 2008 ein regressives souveränistisches Projekt fomiert hat, das auf militaristische Politik zur eigenen Reproduktion angewiesen ist. Außenpolitisch kristallisierte sich diese Regression insbesondere um die Formulierung und versuchte Umsetzung imperialer Verfügungsansprüche über die Ukraine. Erklärungen, die auf das Axiom eines globalen oder russländischen Kapitalismus oder die atavistische Ideologie der russländischen Staatselite rekurrieren, bleiben jedoch defizitär. Alternativ greift der Beitrag auf jüngere historisch-soziologische Zugänge zur Außenpolitik sowie Konzepte der älteren Historischen Sozialwissenschaft in den deutschen Internationalen Beziehungen (IB) zurück und schlägt einen historisierenden Zugang zur Rekonstruktion von Außenpolitik vor. Dieser betont neben der fundamentalen Kontextgebundenheit internationaler Politik die prozesshafte Ko-Konstitution krisenhafter sozioökonomischer Entwicklung, strategischer Elitenprojekte der Herrschaftslegitimierung und Geopolitik.

## Abstract (en)

Russia’s invasion of Ukraine in February 2022 evokes the diagnosis of a war of imperialist conquest. At the same time, *theoretical* approaches to imperialism in the analysis of Russia’s foreign policy enjoy a contradictory niche existence. The critical social sciences, on the one hand, attribute imperialism to capitalist dynamics and US hegemonic aspirations in the 20th and 21st centuries. Political science and sociological approaches, on the other hand, typically focus on the ideological dimension of imperialism or normalize imperialist foreign policies in transhistorical typologies of empire. Based on a critique of existing explanatory accounts, this article attempts to delineate Russian imperialism since the late 2000s. In doing so, it argues that, against the backdrop of a dual crisis of Russia’s political economy of rent and the state elite’s political legitimacy, a regressive sovereignist project has emerged since 2008 that relies on militarist policies for its own reproduction. In Russia’s foreign affairs, this regression crystallized primarily around the articulation and attempted implementation of imperial claims of control over Ukraine’s body politic. However, explanations based on the axiom of a global or Russian capitalism or the atavistic ideology of the Russian state elite remain deficient. Instead, the paper draws on recent historical-sociological perspectives on foreign policy as well as concepts from the older field of Historical Social Science in German International Relations (IB) and proposes a historicizing approach to the reconstruction of foreign policy. Alongside the fundamental contextuality of international politics, this approach emphasizes the processual co-constitution of crisis-ridden socio-economic development, strategic elite projects to (re-)legitimize power, and geopolitics.

„Auferre trucidare rapere falsis nominibus imperium, atque ubi solitudinem faciunt, pacem appellant.“ (Calgacus in Tacitus, Agricola 30, 5)^1^

„Wir haben Mitleid mit dem ukrainischen Volk, das etwas Besseres verdient hat. Wir haben Mitleid mit der ukrainischen Geschichte, die vor unseren Augen zerbröckelt. Und wir bedauern diejenigen, die der Staatspropaganda des Kiewer Regimes […] erlegen sind, die die Ukraine zum ewigen Feind Russlands machen will.“ (Sergei Lawrow, 24.07.2022)^2^

## Russlands Krieg und die Imperialismusdiagnose

Am Morgen des 24. Februar 2022 schlugen die ersten russländischen Raketen auf den Flughäfen in Kyjiw, Lwiw und Charkiw ein.^3^ Auf *deskriptiver *Ebene stand die Diagnose schnell fest: Es handelt sich um die jüngste Eskalation eines russländischen Imperialismus, der nun auch vor klassischen Eroberungskriegen nicht mehr zurückschreckt. Geleitet von der Vorstellung, unter westlichem Druck habe sich die Ukraine zu einem „anti-russischen Projekt“ (Президент Роccии 2022a) entwickelt, erteilte Wladimir Putin seiner Armee den Befehl, das Nachbarland anzugreifen und dessen demokratisch gewählte Regierung durch eine neue Führung von Moskaus Gnaden zu ersetzen. Denn, so Putin, im Kern sei die post-sowjetische Ukraine kein eigener Staat und das Ukrainische eine historische Fehlentwicklung. Bereits Peter der Große habe im 18. Jahrhundert russländische Erde eingesammelt – auch heute dürfe man vor dem Widerstand des Auslands – des „kollektiven Westens“ – nicht zurückschrecken. Ein Land, so Putin (2022b), sei entweder souverän oder eine Kolonie „ohne historische Perspektive“.

Putins Legitimationsfigur ist einerseits imperial und historisch, andererseits souveränistisch und gegenwartsbezogen: Sie folgt einer absoluten, aus einer spezifischen Interpretation der russländischen Geschichte abgeleiteten und übersteigerten Vorstellung politischer, wirtschaftlicher und kultureller Souveränität, die gegen externe Gefahren verteidigt werden müsse (Alles und Badie 2016). Der euphemistisch als „spezielle Militäroperation“^4^ bezeichnete Krieg verfolgt dabei das Ziel, den souveränistischen Anspruch des Kreml auf eine exklusive Einflusssphäre im post-sowjetischen Raum ein für allemal auch gegenüber der Ukraine durchzusetzen (Auer 2015).

Inwiefern können jedoch jenseits der Diagnose imperialer Denkweisen Imperialismus*theorien* zur *Erklärung* der Außenpolitik Russlands und insbesondere der kriegerischen Eskalation des Jahres 2022 beitragen? Kann Russland im Spätputinismus nicht nur im Sinne des imperialen Selbstverständnisses seiner politischen Elite, sondern als Gesellschaftsformation und im Hinblick auf die internen und externen Bedingungsfaktoren seiner Außenpolitik als imperialistisch bezeichnet werden (Geyer 1977; van Herpen 2014)? Russlands Krieg hat derlei Fragen konzeptionell (wieder) ins Licht gerückt, wurde doch mit dem Untergang des Zarenreichs und später mit dem Zerfall der Sowjetunion vorerst das Ende des russischen Imperiums ausgerufen (Cohen 1998, S. 117–149) und einer post-imperialen Transformation (Trenin 2001) das Wort geredet. Russlands Krieg gegen die Ukraine hat jedoch kategoriale Dissonanzen zutage treten lassen. Kann es sein, dass sich die Diagnose eines russländischen Imperialismus oder Kolonialismus als Deskription noch plausibel vertreten lässt, während Imperialismustheorien, die stets ein Bündel an Bedingsfaktoren implizieren, ihr Erklärungspotential verloren haben (Wolfe 1997, S. 388)?^5^

Der Beitrag unternimmt einen Bestimmungsversuch des russländischen Imperialismus seit den späten 2000er Jahren. Er argumentiert, dass sich in Russland vor dem Hintergrund einer Doppelkrise der Rentenökonomie und der politischen Legitimation seit 2008 ein regressives souveränistisches Projekt formiert hat, das sukzessive militaristische Züge angenommen hat. Außenpolitisch kristallisierte sich diese Regression insbesondere um die Formulierung und versuchte Umsetzung imperialer Verfügungsansprüche über die Ukraine. Russlands zweifellos imperialistischer Angriffskrieg gegen die Ukraine im Februar 2022 erschließt sich somit als Ergebnis einer „komplexen Verursachungskette“ (Hellmann 2018) vor dem Hintergrund sich wandelnder nationaler, regionaler und globaler Kontexte. An derem Beginn steht eine bewusste strategische Neujustierung von Herrschaft durch die russländische Staatselite in den Jahren nach der Weltwirtschaftskrise von 2008. Das dahinter stehende politische Projekt hat sich anschließend durch eine Kette „transformativer Ereignisse“ (Sewell Jr. 2005) von 2011–2021 konsolidiert, radikalisiert und – gemessen an den Fehleinschätzungen im Vorfeld der Kriegsentscheidung – pathologisiert. Die russländische Invasion des Jahres 2022 steht demnach nicht einfach in einer linearen historischen Kontinuität militärischer Interventionen in Tschetschenien, Georgien, auf der Krim oder in Syrien. Auch war sie nicht strukturell seit den 1990er-Jahren vorbestimmt. Vielmehr ist sie das Ergebnis einer historisch kontingenten Regression, die sich in letzter Konsequenz vor allem aus innerrussischen Dynamiken ergeben hat. Zu dieser Regression gehören sowohl die Weichenstellungen individueller Akteure der russländischen Entscheidungselite als auch ein „permissiver“ (Soifer 2012, S. 1574–1575) internationaler Kontext, dessen Strukturen selbst jedoch, anders als von (neo-)realistischen AutorInnen behauptet, unzureichend für eine Erklärung des Krieges bleiben.

Der Beitrag skizziert im Folgenden zunächst drei imperialismustheoretische Perspektiven, die sich in der Debatte um Russlands Außenpolitik herausgebildet haben. Deren Erklärungen des Krieges gegen die Ukraine bleiben jedoch ob konzeptionell und empirisch problematischer Annahmen defizitär. Das post-sowjetische Russland entzieht sich den monokausalen und strukturalistischen Bestimmungsversuchen etablierter Imperialismustheorien. Der Beitrag schlägt daher im darauffolgenden Teil einen alternativen Zugang zur Rekonstruktion von Außenpolitik vor. Hierzu wird auf jüngere historisch-soziologische Ansätze der Außenpolitikforschung sowie einen älteren Strang der Historischen Sozialwissenschaft in den deutschen IB zurückgegriffen. Dieser Historisierungsversuch betont neben der fundamentalen Kontextgebundenheit internationaler Politik die prozesshafte Ko-Konstitution von krisenhafter sozioökonomischer Entwicklung, strategischen Elitenprojekten der Herrschaftslegitimierung und Geopolitik. Anschließend rekonstruiert der Beitrag die vertikale und horizontale Regression Russlands seit der Weltwirtschaftskrise von 2008, die in der Kriegsentscheidung einer von personalistischer Vermachtung und souveränistischer Radikalisierung deformierten Staatselite mündete. Der Beitrag schließt mit einer Reflexion der konzeptionell-methodologischen Herausforderungen imperialismustheoretischer Zugänge im Lichte des russländischen Krieges.

## Imperialismustheoretische Perspektiven auf Russlands Außenpolitik

Sowohl der Imperien- als auch der Imperialismus-Begriff erfreuen sich seit den frühen 2000er Jahren ausgesprochener Beliebtheit (Münkler 2005; Harvey 2005; Motyl 2001; Hardt und Negri 2001; Kiely 2010). Insbesondere die Figur des Imperialismus fungiert dabei als eine einflussreiche zeitgenössische hermeneutische Perspektive (Emig 2015). Das Konzept dient nicht nur zur Beschreibung der zaristischen, sowjetischen oder post-sowjetischen Vergangenheit. Auch wird es medial, politisch und (populär)wissenschaftlich zur Erklärung verschiedenster Gegenwartsphänomene herangezogen – von der US-Außenpolitik, Fragen der globalen Ordnung bis zu den revanchistischen Weltbildern von Autokraten (Шишков 2018, S. 24).

Hinter der zunächst unkontrovers anmutenden Diagnose, es handele sich bei Russlands Invasion der Ukraine um einen imperialistischen Krieg, lassen sich sehr unterschiedliche theoretische Perspektiven identifizieren: Russlands Außenpolitik als reaktiver Anti-Imperialismus, Russlands Außenpolitik als „normale“ Politik eines Imperiums und Russlands Außenpolitik als imperialistischer Atavismus. Jede dieser Perspektiven betont unterschiedliche Bündel struktureller Bedingungsfaktoren. Diese scheinen jedoch im Lichte der russländischen Empirie problematisch. Zum einen dominiert in den kritischen Sozialwissenschaften – im starken Unterschied zur Historiographie zaristischer und sowjetischer Imperialismen – ein Imperialismusbegriff, der maßgeblich auf kapitalistische Entwicklungsdynamiken und die globale Hegemonie der Vereinigten Staaten im 20. und 21. Jahrhundert rekurriert (Shor 2010; Harvey 2005). Dies erschwert einen analytischen Zugriff auf die russländische Außenpolitik, erscheint diese somit doch lediglich als Reaktion auf einen global operierenden US-Imperialismus. Die hochgradig gebrochene und in ihrer Reichweite umstrittene kapitalistische Transformation der russländischen Gesellschaft (Ganev 2015) entzieht sich zudem dem Zugriff der meisten klassischen *und* jüngst wiederbelebten kritischen Imperialismustheorien, für die die Annahme verfestigter und krisenhafter kapitalistischer Sozialbeziehungen konstitutiv ist (Milios und Sotiropoulos 2009). Diesen materialistischen stehen politologische und soziologische Änsätze gegenüber, die sich entweder auf ideologische Faktoren wie Weltbilder von Eliten oder intellektuelle Diskurse beschränken oder imperialistische Außenpolitiken als typisches Verhalten von Imperien normalisieren. Dabei werden nicht selten ökonomische Bedingungsfaktoren von Imperialität vernachlässigt (Osterhammel 2022, S. 235). Oder entsprechende Analysen subsumieren qualitativ und historisch sehr verschiedene Modi der ökonomischen und politischen Expansion unter den Begriffen des (intra-kapitalistischen) oder „normalen“ geopolitischen Großmachtkonflikts (Mälksoo 2022, S. 4; Brink 2008; siehe für eine Kritik Teschke und Lacher 2007). Im Folgenden sollen diese drei Perspektiven näher beleuchtet werden.

### Anti-Imperialismus

Eine Gruppe meist marxistisch argumentierender AutorInnen versteht Russlands Außenpolitik als Reaktion auf einen global agierenden US- bzw. westlichen Imperialismus (Buzgalin et al. 2017; Desai et al. 2017; Hudson 2017; für eine entsprechende Reaktion auf den Krieg gegen die Ukraine siehe Marcetic 2022). VertreterInnen dieser Perspektive verstehen Imperialismus in Anlehnung an klassische Imperialismustheorien als globalen Strukturzusammenhang, dem sich Staaten und privatwirtschaftliche Akteure so gut wie nicht entziehen können (für Übersichten siehe Mommsen 1987, S. 27–54; Brink 2008, S. 26–37). Damit verbunden ist die Vorstellung der Vormachtstellung einer kapitalistischen und liberalen US-amerikanischen Supermacht, die, um interne Krisentendenzen zu beheben, auf globaler Ebene imperialistisch und militaristisch auftritt (Harvey 2005). Das entscheidende Kriterium ist hier, dass die Spielregeln internationaler Politik nicht von Russland, sondern von den USA bzw. eines von den USA gesteuerten Konglomerats von Staaten, Organisationen und Institutionen – dem *empire of capital* – festgelegt werden. Russlands gelegentliche „Ausbrüche“ aus diesem System seien vor allem reaktiv (Бузгалин et al. 2016). Die Außenpolitik Russlands erscheint somit entweder als ein Anti-Imperialismus, der *per definitionem* nicht selbst imperialistisch sein kann, oder als ein reaktiver Imperialismus, der jedoch kausal den Widersprüchen des kapitalistisch-imperialistischen Weltsystem untergeordnet bleibt (Ishchenko und Yurchenko 2021).

Unorthodoxere marxistische Kritiker haben diesbezüglich bereits früh moniert, dass die Behauptung, territoriale seien kapitalistischen Expansionslogiken tendenziell untergeordnet (Harvey 2005), eine empirische und keine theoretische Frage sei (Brenner 2006). Diese Kritik wird noch plausibler, berücksichtigt man, dass die Dominanz kapitalistischer Sozialbeziehungen in zahlreichen Gesellschaften jenseits der USA und Europas ebenfalls keine theoretische, sondern eine empirische ist. Aus der Argumentationslogik eines US-zentrierten und kapitalistischen Imperialismus heraus erscheint es nur folgerichtig, dass Russland in zahlreichen Besprechungen des modernen Imperialismus überhaupt nicht oder lediglich als untertheoretisiertes Residuum vorkommt. Vor allem ist dieses Desiderat in Werken auffällig, die explizit die historische und gegenwärtige Vielfalt von Imperialismen rekonstruieren wollen (Kiely 2010).^6^

Politisch bemerkenswert ist, dass sich im Verständnis russländischer Außenpolitik als Anti-Imperialismus die Rhetorik von Putins kleptokratischer Staatselite und die orthodoxe Kapitalismuskritik linker Stimmen treffen. So argumentierte Putin selbst am Tag der Invasion in diese Richtung, freilich ohne den marxistischen Unterbau. In seiner Fernsehansprache schloss er an den stalinistischen Topos des „großen russländischen Volkes“ an, das sich dem westlichen Imperialismus, dem „amerikanischen Imperium der Lügen“, entgegenstellen müsse (Путин 2022). Nicht zuletzt ist es hochproblematisch, dass sozialwissenschaftliche Erklärungen, die diesen Topos übernehmen, die *agency* und damit auch die Verantwortung der russländischen Staatselite für die Kriegsentscheidung abwerten. Damit werden in letzter Konsequenz der Befund eines unilateralen Angriffskriegs sowie die in der Ukraine begangenen Kriegsverbrechen verharmlost.

Neben den politisch-moralischen Problemen entsprechender Theorien hat Alexander Motyl richtigerweise darauf hingewiesen, dass die analytische Schärfe der Imperiums- und Imperialismusbegriffe dadurch verwässert werde, dass insbesondere linke Stimmen sie ausschließlich auf die USA angewendet hätten. So sei es zwar richtig, den US-Imperialismus des 20. und 21. Jahrhunderts zu kritisieren. „Dead wrong“ sei es jedoch, „to define empire and imperialism only in terms of capitalism. This conflation of definitions and causation […] meant that capitalism, and only capitalism, produced imperialism and that, in turn, imperialism was merely its highest stage“ (Motyl 2001, S. 2).

Vergessen scheint heute, dass bereits in den 1970er-Jahren eine Reihe von Studien die strukturalistische Verengung des Imperialismusbegriffs kritisiert hatte (Aron 1974; Krippendorff 1970). Das Wiederaufblühen imperialismustheoretischer Erklärungen der US-amerikanischen Außenpolitik im 20. und 21. Jahrhundert hat diese stark historisierenden und konzeptionell offeneren Zugänge zu imperialismustheoretischen Fragen der Gegenwart jedoch überlagert. Insofern gilt insbesondere für die Analyse nicht-westlicher Imperialismen nach wie vor die 35 Jahre alte Anregung Mommsens (1987, S. 137), nach neuen Formen der Theoriebildung und offenen Erklärungsmodellen jenseits monokausaler Logiken zu suchen.

### Normalisierter Imperialismus

Ein zweites Cluster von Arbeiten stellt Russlands Außenpolitiken in eine Reihe historischer und gegenwärtiger Imperialismen. Zwei Untergruppen können hier identifiziert werden. Eine Gruppe sieht das moderne Russland als normale kapitalistische Großmacht, deren Elite strategisch politische und wirtschaftliche Ziele verfolgt und dabei zuweilen auch militärische Mittel einsetzt (Rees 2017; Cубетто 2015). Diese Perspektive beruht auf der Annahme, dass sich in Russland, wie in anderen westlichen Gesellschaften, kapitalistische Sozialbeziehungen weitestgehend durchgesetzt haben und die Interessenkonstellation zwischen Kapital – ökonomische Expansion – und Staat – territoriale Expansion – im Wesentlichen den westlichen Mustern Ende des 19. bzw. Anfang des 20. Jahrhunderts folgt. Im Kern geht diese Perspektive auf Lenins Kapitalismusbegriff zurück, der Imperialismus als höchstes Stadium kapitalistischer Entwicklung verstand (Lenin [1917] 2016). Der Vorschlag von Anatoli Tschubais (2003) zu Beginn der 2000er Jahre, Russland müsse einen liberalen Imperialismus gegenüber seinen Nachbarstaaten verfolgen, schien Russlands „normalen“ kapitalistischen Großmachtstatus zu bestätigen.

Heute wie zu Lenins Zeiten wird jedoch oft ignoriert, dass Letzterer selbst eine Unterscheidung zwischen einem monopolkapitalistischen und einem militärisch-feudalen Imperialismus traf. Für das zaristische Russland diagnostizierte er gar die Dominanz des Letzteres (Lenin 1915). Seit Lenins Tagen bleibt die Frage, ob und inwiefern Russlands Imperialismus aus kapitalistischen Sozialbeziehungen hervorgegangen ist, beständig im Vagen. An dieser Stelle verweisen einige Autoren auf die semi-periphere Position Russland im kapitalistischen Weltsystem und die in der Folge oft widersprüchlichen Außenpolitiken (Dzarasov 2017). Kritische empirische Untersuchungen zeigen hingegen, dass spätestens seit 2014 die Interessenlagen und außenwirtschaftlichen Strategien des russländischen Staates und der Großunternehmen nicht mehr deckungsgleich sind, ja die staatliche Außenpolitik mittlerweile gegen die Interesses der Unternehmen arbeitet (Matveev 2021).

Für eine zweite Untergruppe der VertreterInnen eines „normalen“ Imperialismus tritt die Frage kapitalistischer Sozialbeziehungen in Russland in den Hintergrund. Ihnen geht es darum, dass Imperien und Großreichsbildungen transhistorische Phänomene sind, die als ein Typ von Herrschaftsbildung neben anderen stehen, etwa Nationalstaaten. Das aus dieser Perspektive resultierende Verständnis geht üblicherweise davon aus, dass die Expansion „ökonomischer, mitunter bloß monetärer, gelegentlich kultureller, häufig militärischer, abschließend fast immer politischer Art“ die Normalform imperialer Außenpolitik sind, wobei „Großreichsbildungen […] prinzipiell das Ergebnis einer aus dem Innern der Herrschaft entspringenden Dynamik [sind], bei der immer weitere Gebiete aufgrund ihrer ökonomischen oder geostrategischen Attraktivität dem expandierenden Machtkomplex eingegliedert werden“ (Münkler 2010, S. 17–18).

Russlands Imperialismus weise spezifische Bedingungsfaktoren auf, die sich trotz politischer Brüche immer wieder reproduzieren würden (Иноземцев/Абалов 2021). Seit Jahrhunderten, so argumentiert etwa der Historiker Alfred Rieber, versuchen zaristische, sowjetische und post-sowjetische Herrscher die vier makrostrukturellen Bedingungen – ökonomische Rückständigkeit, durchlässige Grenzen, eine multikulturelle Gesellschaft und kulturelle Marginalisierung – zu überwinden, die das Land zurückhalten. Das habe zur Herausbildung eines mächtigen imperialen Staatsapparats geführt, der jedoch auf einem fragilen Fundament fuße (Rieber 1994, S. 356). Historisch betrachtet sei der russländische Staat gleichzeitig Kolonisierer, sowohl nach innen als auch außen, und, angesichts seiner Unterentwicklung insbesondere gegenüber dem Westen, Kolonisierter (Etkind 2011). Bereits seit dem 18. Jahrhundert, also lange vor der Existenz der NATO, der EU oder einer globalen US-amerikanischer Hegemonie, hätten diese internen Prozesse Russland periodisch aggressiv nach außen auftreten lassen (Ragsdale 1994, S. 1), so auch gegenwärtig in der Ukraine (Kotkin 2022).

Der Einordnung der gegenwärtigen Außenpolitik Russlands als normalem Imperialismus stehen gleichwohl gewichtige Faktoren gegenüber. So werden zwar staatlicher Expansionsdrang und kriegerische Großmachtpolitik als unausweichliche imperialistische Verhaltensmuster definiert, ohne jedoch konzeptionell und empirisch den diesem Streben zugrunde liegenden Ursachenkomplex erfassen zu können (Motyl 2006, S. 244). Im Zuge des russländischen Angriffskrieges normalisieren insbesondere (neo-)realistische IB-Theorien somit den imperialistischen Unterbau der russländischen Außenpolitik (The Economist 2022). Ferner wurde die Imperialität russländischer Außenpolitik von einigen Studien empirisch bestritten. Eine der wenigen umfassenden empirischen Arbeiten kommt zu dem Ergebnis, dass Russlands Politik gegenüber den post-sowjetischen Nachbarstaaten nicht als neo-imperialistisch, sondern lediglich als hegemonial bezeichnet werden kann und sich somit nicht in bedeutender Weise von Hegemonialbestrebungen anderer Großmächte unterscheidet (Sagramoso 2020). Weitere Untersuchungen streichen zwar die imperialistischen Aspekte russländischer Außenpolitik im Baltikum (Ciziunas 2008) und gegenüber den post-sowjetischen Nachbarn (Orban 2008) heraus, betonen allerdings, dass Russland unter Putin keine ganzheitliche imperialistische Außenpolitik betrieben habe, die auf die tatsächliche Wiederherstellung eines imperialen Raumes gezielt habe.

### Imperialistischer Atavismus

Eine dritte Gruppe versteht den russländischen Imperialismus als Atavismus.^7^ Imperialistische Denkströmungen seien nach 1990 vor allem im Rahmen des wiederauferstandenen Interesses am Eurasianismus in die russländische Diskussion eingezogen (Hausteiner 2010, S. 145–146) und hätten im Zuge der Annexion der Krim immer stärker den politischen Diskurs geprägt (Pain 2016; Kuzio 2017). Die imperialistischen Züge der Moskauer Politik seien dabei atavistisch, da sich das post-sowjetische Russland in einer post-imperialen Situation befinde und das Zarenreich bzw. die Sowjetunion als imperiale Gebilde bereits untergegangen seien (Lieven 2022). Imperiale Denkweisen innerhalb der russländischen Elite und der Bevölkerung stellten ein „imperiales Syndrom“ (Pain 2016) dar, das sich in einen neuen russländischen Nationalismus eingeschrieben habe, jedoch von keiner materiellen Imperialität unterfüttert sei (Kailitz und Umland 2019). BeobachterInnen der partiellen Re-Imperialisierung unter Putin weisen darauf hin, dass es VertreterInnen eines revolutionären Imperialismus zuweilen gelungen sei, Einfluss auf die staatliche Politik zu nehmen, die gleichzeitig jedoch reaktiv geblieben sei (Умланд 2017). Die Politik der herrschenden Elite sei insofern post-imperial, als dass sie nicht auf eine vollständige Restauration des russländischen Imperiums, sondern lediglich auf eine abstrakte Wiederherstellung des einstigen Großmachtstatus ziele (Hanson 2010, S. 175–236). Obwohl Russland ein „normales Land“ sei, werde es von „Menschen mit einer pseudo-imperialistischen Mentalität bevölkert“ (Волошин 2014, S. 380; Übersetzung durch Autor). Im Lichte des Angriffs auf die Ukraine fällt Etkinds Prognose noch pessimistischer aus: „Contemporary Russia, a nation-state, calls itself a federation, like Germany or Switzerland, when in fact it is behaving like an empire in its hour of decline“ (Etkind 2022).

Konzeptionell laufen entsprechende Argumente auf eine Kombination aus imperialer Ideologie und Irredentismus nach dem Zerfall der Sowjetunion hinaus. Das Wiedererstarken populistischer und (neo-)imperialistischer Diskurse seit dem Ende des 20. Jahrhunderts nicht nur in Russland sei der Versuch der Wiederherstellung einer als verloren gegangen erachteten Ordnung (Beissinger 2005, S. 42–45; Frahm und Lehmkuhl 2022). In der Bevölkerung treffe zwar die Vorstellung eines auf globaler Ebene imperial auftretenden Russlands auf breite Zustimmung. Gleichzeitig herrsche jedoch in der Gesellschaft und unter den Eliten ein amorphes Verständnis dessen vor, wo eigentlich die Grenzen Russlands enden und wer eigentlich Teil russländischer Staatlichkeit sei (Волошин 2014, S. 384–385). Im Hinblick auf Russlands Invasion der Ukraine wird betont, dass entsprechende atavistische Vorstellungen von der Staatlichkeit Russlands eng mit der Abwertung der ukrainischen Nation, Kultur, Geschichte und Staatlichkeit verbunden seien und nur in Verbindung mit der Identität als Imperium verstanden werden können. Der Krieg gegen die Ukraine sei damit eine Verteidigung der imaginierten Imperialität russländischer Staatlichkeit, wie sie sich über Jahrhunderte herausgebildet habe (Mälksoo 2022, S. 6).

Bereits vor mehr als 20 Jahren wies Motyl darauf hin, dass sich ein zukünftiges Revival des russländischen Imperialismus auf diese gesellschaftlichen und elitären Ressourcen sowie die relative Stärke des russländischen Staates im Vergleich zu seinen post-sowjetischen Nachbarn stützen werde (Motyl 2001). Tatsächlich wurden in der russländischen Politologie der vergangenen Jahre solche Revitalisierungsversuche offen diskutiert. So sei es für das gegenwärtige Russland und die Phase „nationaler Konsolidierung“ notwendig, „die historischen und kulturellen Besonderheiten Russlands zu berücksichtigen und die Kontinuität mit den historisch vorangegangenen Formen der nationalen Staatlichkeit – dem Russischen Reich und der Sowjetunion – zu wahren“ (Шишков 2018, S. 24–25; Übersetzung durch Autor). Die tatsächliche Wiederherstellung eines Imperiums schien und scheint jedoch wenig wahrscheinlich. Russland fehle vor allem das wirtschaftliche Potenzial und die institutionelle Fähigkeit, Herrschaft jenseits militärischer Repression außerhalb der eigenen Grenzen zu (re)produzieren (Nolte 2010, S. 142).

Trotz unterschiedlicher Nuancierungen besteht unter den drei skizzierten imperialismustheoretischen Perspektiven deskriptive Einigkeit, dass Russlands Außenpolitik in verschiedenerlei Hinsicht imperialistische Charakteristika aufweist. Gleichzeitig ist unbestritten, dass es sich beim post-sowjetischen Russland in materieller Hinsicht nicht (mehr) um ein Imperium handelt. Inwiefern lässt sich jedoch jenseits des Reaktiven – Russlands Außenpolitik als anti-amerikanischer Anti-Imperialismus – und des Atavistischen – Imperialismus als ideologisches Überbleibsel – und der imperialen Normalisierung ein Zugriff auf die Re-Imperialisierung der russländischen Außenpolitik unter Putin formulieren? Eine solche imperialismus *theoretische* Perspektive müsste nicht nur imperiale Denkweisen beschreiben, sondern auch ein Erklärungsangebot für die Herausbildung für Russlands Weg in einen imperialistischen Angriffskrieg liefern können. Dieser Frage, die auf die Erklärung außenpolitischer Regression *innerhalb* der von den oben skizzierten Ansätzen beschriebenen strukturellen Bedingungen zielt, bleiben die meisten Ansätze eine Antwort schuldig. Oder sie greifen auf strukturalistische Annahmen (Kapitalismus, Ideen, historisch persistente Faktoren) zurück, die im Lichte der durchaus vorhandenen historischen Varianz der Außenpolitik Russlands in den vergangenen dreißig Jahren problematisch erscheinen.

Um die Genese des Krieges Russlands gegen die Ukraine sowie die Entscheidung zu einem imperialistischen Feldzug gegen das Wissen und die überwiegende Interessenlage der eigenen Elite, Armee und Gesellschaft zu erklären, reicht demnach keine strukturelle und transhistorische Theorie des Imperialismus (Motyl 2001, S. 12–38). Vielmehr bedarf es eines dynamischen und prozessualen Zugangs, der vor allem auf die *politics* imperialistischer Außenpolitik fokussiert (Doyle 1996, S. 339–349). Hierfür fehlt den oben referierten Perspektiven jedoch das konzeptionelle und methodologische Instrumentarium. Im Folgenden soll daher ein historisch-soziologischer Vorschlag zur Rekonstruktion außenpolitischer Prozesse unterbreitet werden.

## Historische Soziologie als Zugang zu den Außenpolitiken der Imperialismen

In seiner klassischen Studie zum Imperialismus der Zarenzeit fragt Dietrich Geyer auf konzeptioneller Ebene, was die „systeminternen Bedingungen“, die „gesellschaftlichen und ökonomischen Bewegungskräfte“ waren, die die Großmachtpolitik des zaristischen Russlands beeinflussten. Eine solche interne Systemperspektive sei in eine Analyse der „Rückwirkungen des internationalen Engagements auf innerrussische Spannungslagen und Entwicklungsprobleme“ einzubetten. Imperialismus sei als „Reaktion wie als Existenzbedingung staatlicher Machteliten zu verstehen, die unter dem Druck sozialökonomischer Transformationsprozesse ihre Legitimationsbasis schwinden oder doch gefährdet“ (Geyer 1977, S. 11–12) sähen. Geyer stellt folglich, ähnlich wie VertreterInnen der älteren Historischen Sozialwissenschaft in den deutschen IB (Ziebura 1984), die „innere Reformfähigkeit“ von Staatseliten vor dem Hintergrund historisch konkreter sozioökonomischer Problemlagen und internationaler Kontexte in den Mittelpunkt seiner imperialismustheoretischen Rekonstruktion.

Der im Folgenden vorgestellte historisch-soziologische Zugang fußt auf ähnlichen Annahmen. Auch er geht davon aus, dass imperialistische Projekte und ihre in letzter Konsequenz meist kriegerischen Außenpolitiken ihren Ausgangspunkt in krisenhaften Sozialformationen und historisch spezifischen internationalen Kontexten haben (Wolfe 1997, S. 397–399). Der Zusammenhang zwischen Krise und Krieg ist jedoch kein linearer und zeitlich synchroner. Auf Krisen – das heißt: in der Auseinandersetzung mit strukturellen Brüchen und Legitimitätskrisen von Ordnung – reagieren Eliten mit neuen, oft kreativen politischen Projekten. Imperialismus, „the process of establishing and maintaining an empire“ (Doyle 1996, S. 19), kann eine solche Reaktion sein. Die Re-Imperialisierung der russländischen Außenpolitik unter Putin, mit der bisher weitreichendsten Entscheidung eines umfänglichen Eroberungskrieges, kann als ein solcher Prozess verstanden werden.

Um diesen Prozess zu rekonstruieren, greift der Beitrag auf jüngere Versuche zurück, Konzepte und Methodologien der Historischen Soziolgie für die Analyse internationaler Politik fruchtbar zu machen (Hoppe 2021). Diese betonen die Ko-Konstitution sozioökonomischer Entwicklung, strategische Elitenstrategien der Herrschaftslegitimierung und Geopolitik. Im Mittelpunkt steht der Fokus auf die sozioökonomischen Ursprünge, politische Formierung und historische Kontextgebundenheit strategischer Projekte, mit denen staatliche Eliten versuchen, ihr lokales, nationales oder internationales Umfeld zu reproduzieren oder zu verändern. Methodisch zielt ein solcher Ansatz auf die Rekonstruktion mit- und gegeneinander agierender und stets kontestierter strategischer Projekte, die in historisch und räumlich unterschiedlichen Kontexten von staatlichen Eliten formiert und umgesetzt werden (Teschke und Lacher 2007; Teschke und Cemgil 2014; Schlichte 2012, 2017; Hoppe 2021; Hellmann 2018). Der Begriff des Projekts betont dabei die aktive Konstruktion entsprechender Elitenstrategien durch historisch spezifische Akteure, während der Fokus auf Interaktion und unintendierte Konsequenzen den Prozesscharakter der Formierung und Umsetzung entsprechender Strategien gegen oder in Kooperation mit anderen Projekten herausstreicht. Imperialismustheoretisch knüpft diese Perspektivverschiebung – weg vom antezedenten Strukturzusammenhang von Außenpolitik, hin zu ihrem Prozesscharakter – an Vorschläge an, historische und gegenwärtige Imperialismen als Projekte zu konzeptualisieren, die über einen längeren Zeitraum von Elitenfraktionen vorangetrieben werden (Kiely 2010, S. 4, 253).

Heuristisch können die Versuche staatlicher Eliten, strategische Projekte zu konstruieren, in ihrer vertikalen und horizontalen Dimension erfasst werden (Hoppe 2021, S. 50–57). Vertikal entwickeln Eliten diese Projekte, um sozioökonomische Konflikte zu stabilisieren, zu verändern oder zu überwinden und Herrschaft (neu) zu legitimieren. Horizontal antizipieren (staatliche) Eliten die Projekte anderer (staatlicher) Eliten. Eine Historische Soziologie der internationalen Politik geht davon aus, dass die Art und Weise, wie Staatseliten diese vertikalen und horizontalen Dimensionen wahrnehmen und kreativ „bearbeiten“, nicht vollständig aus strukturellen Bedigungsfaktoren auf internationaler, staatlicher oder individueller Ebene abgelesen werden kann. Aus diesem Umstand resultiert die forschungspraktische und methodologische Selbstverpflichtung, „to always analyse from the perspective of agency […] by us[ing] history itself as a framing device“ (Knafo und Teschke 2020, S. 70–71). Die in den Blick genommenen strategischen Elitenprojekte müssen folglich historisch rekonstruiert und gegen bestehende theoretische oder ideologische Reifizierungen abgewogen werden.

Die damit beschriebene historisch-soziologische Methodologie entspricht einem abduktiven Ansatz (Hoppe 2021, S. 51): eine Strategie des praktischen Schließens, die die kreative Problematisierung, Verwerfung oder Weiterentwicklung bestehender Theorien im Lichte des historischen Erwartungs- und Möglichkeitshorizonts umfasst (Herborth 2013, S. 350; Sturm 2006). Die Prinzipien der Historizität und der Abduktion bedeuten jedoch keinen theorie- und generalisierungsfreien Empirismus. Im Gegenteil ist eine Historische Soziologie der internationalen Politik notwendigerweise auf Theorien oder, allgemeiner ausgedrückt, praktische und theoretische Reifizierungen angewiesen, die den Akteuren (und diese sich selbst) strukturelle Zwänge auferlegen (Osterhammel 2016, S. 24). Der daraus resultierende Modus der Theoriebildung kann als historisierende Problematisierung bezeichnet werden (Teschke und Lacher 2007, S. 570–571).

Das vorgeschlagene historisch-soziologische Vorgehen unterscheidet sich nicht nur fundamental von strukturalistischen Imperialismustheorien, sondern auch von gängigen theoretischen Zugängen zur Außenpolitik, die entweder auf individuelle oder Gruppenentscheidungen oder strukturelle Bedingungsfaktoren fokussieren. Die hier vertretene Perspektive ist hingegen inspiriert von historiographischen Zugriffen auf staatliche Strategiebildung, die vor allem deren Prozesscharrakter betont (Murray et al. 1994). Eine solche Methodik schließt trotz der beschworenen Historizität keineswegs aus, *gegenwärtige* Ereignisse und strukturelle Brüche internationaler Politik in den Blick zu nehmen. Historisch heißt in diesem Sinne weder „abgeschlossen“ noch „in der Vergangenheit liegend“. Historisierung zielt darauf ab, Außen- und internationale Politik als historisch ergebnisoffenen – im Gegensatz zu strukturell vorbestimmten – Prozess zu begreifen, der die kontextsensible Rekonstruktion von Handlungsketten erfordert (Teschke und Cemgil 2014; Hoppe 2021, S. 51–54; Hellmann 2018, S. 85). Die folgende empirische Annäherung wird daher insbesondere auf Chronologie, Akteursstrategien und Kontextveränderungen achten.^8^

## Russlands Regression, 2008–2022. Eine Historisierung

Im Folgenden wird argumentiert, dass Russlands Feldzug gegen die Ukraine seit Februar 2022 Teil und vorläufiger Höhepunkt eines regressiven Prozesses der sukzessiven Militarisierung der Außenpolitik ist. Hinter der Regression steht ein politisches Elitenprojekt, das sich insbesondere in den Jahren 2008 bis 2012 fomierte und als Souveränismus bezeichnet werden kann. Das Projekt reagierte einerseits auf interne politökonomische und Legitimitätskrisen und andererseits auf geopolitische Ereignisse, die sich retrospektiv als transformativ erwiesen haben.

*Souveränismus* bezeichnet den politischen Anspruch, einerseits uneingeschränkte Entscheidungsmacht auszuüben und sich andererseits von einer als extern und illegitim wahrgenommenen Autorität zu emanzipieren (Alles und Badie 2016, S. 6).^9^ In der Praxis äußert sich dieser Anspruch in Form von Politiken, die die ökonomische, kulturelle und politische Unabhängigkeit auf Kosten anderer Ziele überbetonen und versuchen umzusetzen. Unter *Militarismus* soll ein Projekt der Aufwertung des Militärischen in Politik, Wirtschaft und Gesellschaft sowie die Überzeugung und Bereitschaft, das Militär als eines der bevorzugten politischen Mittel einzusetzen, verstanden werden (Mabee und Vucetic 2018). Als *regressiv* bezeichne ich den dargestellten Prozess, da die Außenpolitik Russlands seit den späten 2000er Jahren durch den zunehmenden Rückgriff auf das Militär innen- und außenpolitisch immer höhere politische, wirtschaftliche und soziale Kosten in Kauf genommen sowie zur De-Institutionalisierung der Politik und Primitivisierung der politischen Ökonomie geführt hat, ohne dass die eigenen Ziele in der internationalen Politik erreicht wurden.^10^

Der Begriff der Regression bedarf dabei der Begründung, schließlich sind imperiale Denkfiguren und Herrschaftsansprüche seit Ende der 1990er-Jahre in der russländischen Politik präsent. Die Figur des Widerstandes gegen den Faschismus etwa, die von Putins Staatselite absurderweise auch heute zur Legitierung des militärischen Vorgehens gegen die Ukraine vertreten wird, fand bereits in den russländischen Militärinterventionen der 1990er-Jahre Anwendung. Der damalige Militärgeneral und spätere Präsidentschaftskandidat Alexander Lebed begründete 1992 Russlands Eingreifen in den Konflikt um die Republik Moldau mit dem Kampf gegen ein faschistisches Regime (Lebed 1997). Handeltes es sich damals jedoch um Verlautbarungen einzelner Akteure, werden derlei Argumente heute in den Rang offizieller diplomatischer Stellungnahmen erhoben. Die Bereitschaft und Fähigkeit der Putinschen Entscheidungselite, einen imperialistischen Angriffskrieg der Größenordnung des Februars 2022 zu führen, war weder bereits in den frühen 1990er-Jahren noch Ende der 2000er-Jahre – trotz Putins revisionistischer Rede auf der Münchner Sicherheitskonferenz 2007 und einer Militärintervention in Georgien im August 2008 – in dieser Form absehbar. Regressiv steht in diesem Sinne nicht zuletzt für das Ungleichgewicht zwischen einem immer absoluter vorgetragenen imperialen Anspruch und der abnehmenden Fähigkeit zur Durchsetzung dieses Anspruchs. Dazu zählt auch das Unvermögen, die tatsächlichen Kosten und Konsequenzen des eigenen Handelns adäquat einschätzen zu können.

Der vorgestellten Heuristik folgend rekonstruiert der Beitrag zunächst die vertikale Neujustierung der Herrschaft, die die russländischen Staatselite um Putin zwischen 2008 und 2012 vollzog. Anschließend wird gezeigt, wie das in diesem Zuge geformte souveränistische Projekt vor dem Hintergrund mehrerer konfliktiver internationaler Kontexte geopolitisch aufgeladen wurde. Schlussendlich wird die Re-Imperialisierung der russländischen Politik in den horizontalen Kontext der Entwicklungen gestellt, die sich seit Ende 2013 in und um die Ukraine vollzogen. Ähnlich wie Geyers (1977) Studie zum Imperialismus der Zarenzeit wird dabei versucht, das Wechselspiel zwischen internen (vertikalen) und externen (horizontalen) Bedingungsfaktoren sowie Akteursstrategien in den Blick zu nehmen.

### Krise des russländischen Rentierismus und vertikale Regression, 2008–2012

Die Invasion des Jahres 2022 bleibt unverständlich, wenn man sie lediglich in die Reihe horizontaler militärischer Konflikte – Moldau 1992, Tschetschenien 1994–96 bzw. 1999–2009, Georgien 2008, Krim und Ostukraine 2014 sowie Syrien 2015 – stellt, in denen Russland seit den 1990er-Jahren aktiv war. Die Fehlkalkulation und das Ausmaß der Selbstschädigung des Krieges gegen die Ukraine werden nur verständlich, wenn man das Muster regelmäßiger Militärinterventionen in den Kontext der neuartigen vertikalen Regression stellt, die Russlands politische Ökonomie seit der Weltwirtschaftskrise von 2008 kennzeichnet.

Diese politische Ökonomie ist maßgeblich um die Extraktion und den Export von Rohstoffen, insbesondere Öl und Gas, und das Management der hierdurch generierten Renten strukturiert. Mit dem Ziel, diese einseitige wirtschafltiche Ausrichtung zu überwinden, hatte Putins Nachfolger Dmitri Medwedew 2008 zunächst eine wirtschaftsliberale Modernisierung in Aussicht gestellt – wenn auch innerhalb der autoritären Strukturen des russländischen Staates (Shevtsova 2010). Anfang der 2010er Jahre kam dieses Projekt mit der Entscheidung Putins, zum dritten Mal die Präsidentschaft zu übernehmen, zum Erliegen. Die Konsequenzen der Rückkehr Putins reichten weit über die für elektorale Autokratien üblichen personellen Neubesetzungen hinaus. Sie waren Ausdruck einer umfassenderen Neugestaltung der Beziehungen zwischen Staat, Gesellschaft und Wirtschaft. Das souveränistische Projekt, das sich in den Jahren des Interregnums Medwedews herausbildete und ab 2012 konsolidierte, adressierte zwei interne Krisenkomplexe: die Erschöpfung eines auf Rentenextraktion basierenden sozioökonomischen Wachstumsmodells sowie die herrschaftsdestabilisierende Erosion politischer Legitimität.

Die sozioökonomische Erschöpfung Ende der 2000er-Jahre ergab sich aus der verpassten Chance, im Verlauf der ersten beiden Präsidentschaften Putins (2000–2008) eine umfangreiche Reform des russländischen Rentenmanagementsystems (RMS) durchzuführen, „a characteristic set of institutions and mechanisms by which […] rents are produced, collected, and redistributed throughout the economy“ (Gaddy und Ickes 2015, S. 11). Trotz einer Zunahme des Bruttoinlandsproduktes (BIP) um 83 % zwischen 2000 und 2008, der Produktivität um 70 % und der Investitionen in Anlagekapital um mer als 100 % (Kudrin und Gurvich 2015, S. 30), verging fast ein Jahrzehnt, ohne dass die Konzentration auf die Rohstoffsektoren verringert wurde. Wachstumsimpulse jenseits der Öl- und Gasindustrie rührten maßgeblich von der massiven Rubelabwertung 1998 oder waren eine indirekte Folge der Konjunktur der Öl- und Gasbranche. Vor diesem Hintergrund sollte ins Bewusstsein gerufen werden, dass insbesondere die seit Beginn der 2010er Jahre initiierten westlich-russländischen Energiegroßprojekte, wie die Nord Stream-Pipelines oder Beteiligungen europäischer Unternehmen an neuen LNG-Projekten, zur Stabilisierung des russländischen Rohstoffextraktivismus und somit zur Reproduktion der den Putinismus stützenden politischen Ökonomie beigetragen haben.

Gleichzeitig erreichten sowohl Putins Macht als auch seine Popularitätswerte im Jahr 2008 ihren vorzeitigen Höhepunkt. Niemals zuvor in der russländischen oder sowjetischen Geschichte stieg der messbare Wohlstand der Bevölkerung so schnell und stetig wie in seinen ersten zwei Amtszeiten. Die durchschnittlichen Haushaltsnettoeinkommen stiegen um 140 %, der Durchschnittslohn lag 2008 bei über 1000 $. Der Anteil derer, die unterhalb der Armutsgrenze leben, d. h. täglich weniger als 10,80 $ (Stand 2012) zur Verfügung hatten, sank von 29 % im Jahr 2000 auf 13 % im Jahr 2011. Technologische Revolutionen im Alltagsleben unterstrichen das Gefühl einer ungekannten Wohlstandsdekade. So lag 2011 der Anteil der russländischen Bevölkerung mit Mobiltelefonen höher als in Frankreich, Japan oder den USA. Zur selben Zeit besaßen 60 % der Stadt- und 46 % der Landbevölkerung einen Computer, während es im Jahr 2000 durchschnittlich nur 25 % waren. Ein neues Konsumversprechen in Form von Einkaufszentren, Supermärkten und Kinos machte sich auch in Russlands kleineren Städten breit (Dmitriev und Treisman 2012, S. 66–67). Neben der Rubelabwertung 1998 und den hohen Weltmarktpreisen für Öl insbesondere seit 2003 waren für den Wirtschaftsaufschwung durchaus auch Errungenschaften aus Putins erster Amtszeit verantwortlich. Hierunter zählen etwa die relative Stabilität innerhalb der Elite, ein überholtes Steuer- und Arbeitsrecht und kleinere juristische und bürokratische Reformen (Balzer 2003, S. 212).

Die vertiefte Integration in die Weltwirtschaft sowie wirtschaftliche und bürokratische Teilreformen führten jedoch nicht zu einer tiefgreifenden Umgestaltung des RMS. Folglich traf der Ausbruch der globalen Finanzkrise 2008 und der damit einhergehende massive Einbruch der weltweiten Rohstoffpreise Russland hart – zur Überraschung der Staatselite, die fast ein Jahrzehnt hoher verteilbarer Renteneinkommen gewohnt war. Allein 2009 schrumpfte Russlands Wirtschaft um 7,9 %, der höchste Wert unter den G20-Ländern. Die Industrieproduktion brach um 24 % ein, die Inflation erreichte 8,8 % (Sutela 2012, S. 2018). Insbesondere der starke Verfall der Weltmarktpreise für Öl und Gas und das Versiegen der verteilbaren Renten legte ökonomische Probleme und, in der Folge, politische Spannungen zwischen verschiedenen politischen Clans offen. Als entscheidend entpuppte sich der Konflikt zwischen zu diesem Zeitpunkt noch als „liberalen Reformern“ bezeichneten Eliten und konservativen Etatisten, aus dem schließlich letztere als Sieger hervorgehen sollten.

Vor diesem Hintergrund kristallisierte sich eine zweite Krise heraus, die die Erosion politischer Legitimität betraf. Die Krise schlug sich im Winter 2011/12 in den größten Massendemonstrationen der post-sowjetischen Geschichte nieder. Die Folgen der Weltwirtschaftskrise von 2008 gaben auch die Konturen der politischen Krise drei Jahre später vor, indem sie die Zusammensetzung der Protestierenden präkonfigurierten. So erschütterte die Krise die ökonomische Geographie Russlands, die sich seit Ende der 1990er-Jahre herausgebildet hatte. Unter den rund 150 russländischen Städten mit 50.000–250.000 Einwohnern (etwa 30 % der Gesamtbevölkerung) befinden sich zahlreiche sogenannte Monostädte. Diese Städte sind oft auf nur einen Sektor oder Arbeitgeber ausgerichtet (Crowley 2016). Sie bestehen einerseits aus Unternehmen, die für den Binnenmarkt produzieren (etwa im Maschinenbau, der Lebensmittelindustrie oder dem Rüstungssektor) und sich durch ein niedriges Lohnniveau auszeichnen. Zum anderen gibt es Monostädte der gleichen Größe, die oft ebenfalls nur von einem Industriezweig abhängig, aber stark exportorientiert sind, wie z. B. die öl- und gasproduzierenden Unternehmen in der Region Tjumen, die durch den Einbruch der weltweiten Rohstoffpreise 2008 von Finanzierungsquellen zu Subventionsempfängern wurden. Der Kreml sah sich demnach mit einem Schwinden von Renten konfrontiert, die bis dato zentral für die Reproduktion des politökonomischen status quo und der Erzeugung politischer Legitimität gewesen waren.

Die Auswirkungen dieser seit 2008 schwelenden Krise des RMS auf ihren Machterhalt bekam Russlands herrschende Staatselite erstmals bei den Dumawahlen 2011 zu spüren. In zahlreichen Monostädten – seit 2000 eigentlich die tragenden Säulen des von Putin etablierten politischen Systems – lag das Ergebnis der hegemonialen Staatspartei „Einiges Russland“ bei 29–38 % – und damit auf dem Niveau der Großstädte, die bis zu diesem Zeitpunkt als Zentren des Protests galten (Zubarevich 2016). Der politische Funke der Winterproteste 2011/2012 waren insbesondere die massiven Fälschungsvorwürfe im Zusammenhang mit der Dumawahl vom 4. Dezember 2011 und die zuvor angekündigte erneute Kandidatur Putins für eine dritte Amtszeit als Präsident. Die durch die Rochade zwischen Putin und Medwedew symbolisierte Verkrustung des politischen Systems stieß insbesondere unter der großstädtischen Mittelschicht, bei jungen und urbanen Bevölkerungsschichten sowie Akteuren aus dem Kunst- und Kulturbereich auf Widerstand. Die Proteste wurden zunächst von einer losen Koalition aus Liberalen, Linken und Nationalisten getragen. Später schlossen sich MedienvertreterInnen, Popstars und ehemalige SchauspielerInnen aus dem politisch-kulturellen Establishment an. Den OrganisatorInnen der Proteste gelang es jedoch zu keinem Zeitpunkt, aus den primär sozioökonomischen Forderungen der Regionen und den postmaterialistischen und partizipatorischen Forderungen der Großstädte eine einheitliche Oppositionsbewegung zu formen. Gleichwohl war es aus der Perspektive der staatlichen Eliten beunruhigend, dass sich die Proteste über das ganze Land ausbreiteten, einschließlich kleiner und mittlerer (Mono‑)Städte in den Regionen, wobei die größte Demonstration am 24. Dezember 2011 in Moskau mit rund 120.000 Teilnehmern stattfand (Chehonadskih 2014, S. 196–200).

Putin war anno 2011 folglich sowohl mit einer sozioökonomischen und einer Legitimitätskrise als auch sozialer und politischer Instabilität in Form eines von den Protestierenden repräsentierten demokratisch-nationalistischen Projektes konfrontiert (Pain 2016, S. 46). In dieser *critical juncture *stand die Staatselite vor der Wahl zwischen progressivem Reform- oder regressivem Gegenprojekt.

### Souveränisierung und Militarisierung von allem. US-Strategie, Arabischer Frühling, und der Aufstieg Chinas

Putins innerer Zirkel entschied sich für eine regressive „hegemoniale Intervention“ (Valenza 2022, S. 3). Diese forcierte einen patriotisch-nationalistischen und konservativen Identitätsdiskurs. Zwar wurden auch weiterhin sozioökonomische Entwicklungsziele proklamiert, etwa 2012 die sog. Mai-Dekrete, allerdings wurden diese dem neuen politischen Projekt der souveränistischen Vergemeinschaftung untergeordnet. Das regressive staatliche Gegenprojekt erfüllte zwei Funktionen: die Sicherung eines personalistischen Herrschaftssystems, dessen Schaltstellen in zunehmendem Maße vom obersten Patron Putin und einem Netz loyaler Sub-Patrone besetzt wurde, und die Versiegelung des Herrschaftssystems gegenüber externen Einflüssen. Die Re-Imperialisierung und Militarisierung der Außenpolitik sollte später auf diesem Fundament aufbauen und zu einer zentralen Stütze der Reproduktion des Regimes werden.

Das neue Herrschaftsprojekt zielte innenpolitisch auf die Spaltung bzw. Verhinderung einer nationalen und koordinierten politischen Oppositionsbewegung. Im Unterschied zu den Jahren 2000–2008 gaben Putin und die mit der Kampagne betrauten IdeologInnen die urbanen und einem westlichen Lebensstil zugeneigten Milieus der Großstädte politisch auf. Stattdessen beschränkte sich die Staatselite nun darauf, die Unterstützung der monoindustriell geprägten Provinzstädte und der wirtschaftlich abgehängten ländlichen Regionen zu sichern, vor allem durch höhere staatliche Zuwendungen (Zubarevič 2012).

Ferner versuchte die Kampagne ideologisch, die Verantwortung für die bestehende sozioökonomische Krise und die Erosion der eigenen Legitimität nach außen abzuleiten. Zentral hierfür war die Propagierung der konservativen Figur der politisch-sozialen Stabilität, die die Angst vor einer „Bedrohung der nationalen Sicherheit“, der „territorialen Integrität“ und der „russischen Kultur, Mentalität und Spiritualität“ betonte. Für Lew Gudkow (2015, S. 24), Direktor des Moskauer Lewada-Zentrums, hat dieser Diskurs die Gestalt einer „kollektiven Metaphysik“ angenommen, die um die „ewige Konfrontation zwischen Russland und dem Westen als jeweils eigenständige und geschlossene Zivilisationen“ strukturiert ist. Der Zugriff auf und das Management von Renten und die „Produktion von Sicherheitsrisiken“ und deren Kommunikation „nach oben“ hängen dabei eng zusammen. In Russlands stark hierarchisiertem und personalistischem Herrschaftssystem ist die bürokratische Ressourcenallokation maßgeblich eine Funktion der vom obersten Patron Putin wahrgenommenen Gefahrenlage (Кордонcкий 2007). Politische, bürokratische und wirtschaftliche Akteure haben demnach einen materiellen Anreiz, die Ideologie eines ewigen Konflikts mit dem Westen *von unten* zu reproduzieren. Der regressive Charakter und die relative Stabilität des Putinschen Souveränismus ergeben sich vor allem aus dieser „Ko-Konstruktion“ des Systems durch Staat, Wirtschaft und Gesellschaft (Robertson und Greene 2017).

Geopolitisch aufgeladen wurde das Projekt entlang mehrerer internationaler Konflikte, die zuweilen auch innenpolitisch konfrontativ ausgetragen wurden. Zwar waren bereits Putins Rede auf der Münchner Sicherheitskonferenz 2007 sowie Russlands zwölftägiger Augustkrieg gegen Georgien im Jahr 2008 Anzeichen einer Abkehr von Putins vergleichsweise kooperativen ersten Jahren als Präsident. Auch ist zutreffend, dass die zunehmend konfrontativere Haltung Russlands durchaus an bereits vorhandene Strömungen im außenpolitischen Denken der Staatselite anknüpft. Russland war zu diesem Zeitpunkt jedoch nach wie vor diplomatisch von westlichen Staaten ansprech- und einhegbar (Allison 2013, S. 150–169). Zwischen 2008 und 2012, im Unterschied zu Putins ersten beiden Amtszeiten, radikalisierte sich jedoch die Einstellung gegenüber den USA und der NATO. In der im Februar 2010 vorgestellten Militärdoktrin Russlands (Президент Роccии 2010) wurde die Gefahr der Ausweitung und globalen Ambitionen der NATO erstmals an die erste Stelle potenzieller militärischer Bedrohungsszenarien gesetzt.

Die erhöhte Salienz wahrgenommener „westlicher Bedrohungen“ muss ihrerseits vor dem Kontext der US-amerikanischen Militäreinsätze in Afghanistan und Irak sowie des gegen den Widerstand Deutschlands und Frankreichs gescheiterten Versuchs der Bush-Regierung im Jahr 2008 gesehen werden, Georgien und die Ukraine in die NATO aufzunehmen. Ein permissiver internationaler Kontext trug somit ohne Frage zur geopolitischen Aufladung des Putinschen Souveränismus bei. Ob ein entschiedeneres militärisches Auftreten des Westens bereits zu diesem Zeitpunkt die Militarisierung Russlands gebremst oder beschleunigt hätte, ist zwar eine politisch interessante Frage. Sie bleibt letztlich jedoch kontrafaktische Spekulation.

Die Souveränisierung und Militarisierung der russländischen Politik erfolgte nicht ohne innenpolitische Konflikte, die Risse innerhalb der Elite und damit auch eine relative Kontingenz des neuen spätputinschen Projektes offenlegten. So enthielt sich Medwedew bei der Abstimmung im UN-Sicherheitsrat über die Resolution 1973 der Stimme. Ziel der Resolution war die Einrichtung einer Flugverbotszone über Libyen. Der innenpolitische Konflikt um die Entscheidung, die von Putin als Schwäche gegenüber dem „kollektiven Westen“ gewertet wurde, erwies sich als besonders folgenschwer und wurde zum Kristallisationspunkt des sich herausbildenden regressiven Projekts. Im Zuge der westlichen Militäraktionen, die auf die Umsetzung der Resolution folgten, wurde der libysche Präsident Muammar Gaddafi von bewaffneten Gruppen gelyncht. Letztere hatten zuvor die Koordinaten des Aufenthaltsortes Gaddafis vom französischen Militär erhalten. In den Wochen nach der Stimmenenthaltung Medwedews und der anschließenden weiten Auslegung des Mandats seitens der westlichen Allianz (Allison 2013, S. 191–192) brach zwischen Medwedew und Putin ein zuweilen öffentlich ausgetragener Konflikt aus. Während eines Besuchs in einer Fabrik für ballistische Raketen erklärte Putin, zu dieser Zeit Ministerpräsident, dass westliche Forderungen nach einer bewaffneten Intervention in Libyen „mittelalterlichen Aufrufen zu Kreuzzügen“ ähnelten. Russland gebe dies das Recht, sein Militär umfangreich aufzurüsten. Von Medwedew wurden entsprechende Einlassungen Putins zurückgewiesen – derlei öffentliche Äußerungen seien „inakzeptabel“ (Cмирнов 2011).

Die zu diesem Zeitpunkt etablierte Elitenkonstellation in Russland, mit einem insgesamt schwachen Präsidenten Medwedew, der zudem seit 2009 mit einem sog. *reset/peresagruska* mit den USA beschäftigt war, dabei jedoch von seinem im Hintergrund operierenden Patron Putin kontrolliert und kritisiert wurde, war widersprüchlich und paradox. Auf Putins außenpolitische Agenda und militaristische Radikalisierung in seiner dritten und vierten Amtszeit sollte sein sich in dieser Zeit verhärtender Anti-Amerikanismus und die auch durch die Geheimdienstsozialisation verstärkte, habituelle Angst vor einem Sturz von außen große Auswirkungen haben. Russlands Bereitschaft zu einer militärischen Intervention in Syrien im September 2015 zur Verhinderung eines weiteren *regime change* ist maßgeblich aus der Melange aus interner Krise und externer Libyen-Erfahrung erklärbar. Innenpolitisch führte der Konflikt zudem dazu, dass Putin die russländische Politk noch stärker personalisierte, um die Entstehung semi-autonomer Entscheidungspole zu verhindern.

Mehrere programmatische Artikel, die Putin zwischen Mitte Januar und Ende Februar 2012 in führenden russländischen Zeitungen veröffentlichte, verdeutlichen die ideologischen Kernpunkte des souveränistischen Projektes, das sich seit Anfang der 2010er Jahre in immer kohärenterer Form herausbildete. Die Beiträge fungierten als ideologisch-strategische Rahmung von Putins Präsidentschaftskampagne und berührten ein breites Spektrum von Themen – von der Innenpolitik über die Wirtschaftsstrategie bis hin zu kulturellen Fragen und internationaler Politik. In einem langen Artikel mit dem Titel „Wir brauchen eine neue Wirtschaft“ zeichnete Putin (2012a) zunächst noch ein sehr nüchternes Bild von den Problemen der russländischen Ökonomie. Um diese anzugehen, argumentierte er, müsse man sich an den asiatischen Entwicklungsstaaten orientieren. Für Putin stand die Chiffre Asien vor allem für den Aufstiegs Chinas, den er als nicht-westliche Chance für Russland auffasste. Die 2008 eingesetzte Weltwirtschaftskrise spielte auch hier eine zentrale Rolle. Die Krise zeige, so Putin, dass sich die weltpolitische Balance zukünftig nach Asien und insbesondere nach China verlagere. Sowohl die USA als auch die Europäische Union (EU) und damit die bisherigen Zentren der Weltwirtschaft hätten mit massiven Auswirkungen der Krise auf ihre Volkswirtschaften zu kämpfen, während sich China als globaler Stabilisator erweise. Aus dieser Einschätzung erwuchs in den darauffolgenden Jahren eine auch öffentlich massiv propagierte „Wende nach Osten“ (Hoppe und Rogova 2020). Diese besteht aus verschiedenen regionalen Entwicklungs- und Außenwirtschaftsstrategien, die darauf zielen, die wirtschaftliche und infrastrukturelle Lage des russländischen Fernen Ostens (RFO) zu verbessern und Russland in den asiatisch-pazifischen Wirtschaftsraum zu integrieren. Im Zuge seiner Präsidentschaftskampagne 2012 forderte Putin (2012c) konsequenterweise, „den chinesischen Wind in den Segeln unserer Wirtschaft einzufangen“.

Im sich neu formierenden Putinschen Projekt sollten Wirtschafts‑, Außen- und Militärpolitik enger als zuvor verzahnt werden. In einem programmatischen Artikel in *Moskowskije Nowosti* erläuterte Putin, dass „die Außenpolitik ein integraler Bestandteil jeder nationalen Strategie“ sei und „externe Herausforderungen und die sich verändernde Welt die Wirtschafts‑, Kultur‑, Steuer- und Investitionspolitik beeinflussen“ würden (Путин 2012c). Ein weiterer Beitrag in der *Rossiiskaya Gazeta* im Februar 2012 (Путин 2012b) argumentierte, dass „Russlands sich [u]nter den Umständen [einer Welt im Umbruch] nicht allein auf diplomatische und wirtschaftliche Methoden verlassen kann, um Konflikte zu lösen. Unser Land steht vor der Aufgabe, sein militärisches Potenzial im Rahmen einer Abschreckungsstrategie ausreichend zu entwickeln.“ Putin unterstrich hier bereits die entscheidende Rolle des Militärs, das „eine unabdingbare Voraussetzung sei, dass sich Russland sicher fühlt und dass unsere Partner den Argumenten unseres Landes Gehör schenken“. Das Militär sollte die maßgebliche Garantie dafür sein, dass Russland von anderen überhaupt erst wahrgenommen und der angestrebte Entwicklungsweg des Landes abgesichert werde.

Die auf Russisch verfassten Artikel richteten sich in erster Linie an die eigene Bevölkerung. Sie legten die Eckpunkte eines insbesondere gegen äußere Feinde gerichteten Projektes offen. Dieser Souveränismus ging über einen nach innen gerichteten herrschaftsstabilisierenden Nationalismus, Patriotismus oder Konservatismus hinaus und wies mit der Ablehnung westlicher Hegemomie, der anvisierten Partnerschaft mit China sowie der zentralen Rolle der Armee umfangreiche außen- und militärpolitischen Dimensionen auf.

Für die imperialismustheoretische Verortung der Außenpolitik unter Putin ist es wichtig zu verstehen, dass sich das der Re-Imperialisierung zugrunde liegende, strategische Projekt im Zusammenspiel multipler interner *und* externer Krisen herausbildete und weder allein auf wirtschaftliche oder ökonomische (erst recht jedoch keine kapitalistischen) Strukturen oder ein bereits seit den 1990er-Jahren prädeterminiertes Denken Putins zurückzuführen ist.

### Die „Ukraine-Frage“: Radikalisierter imperialistischer Atavismus und horizontale Regression

Die Entscheidung Putins für eine umfängliche Invasion der Ukraine gegen das Wissen und die Interessen eines Großteils der eigenen Elite, Bevölkerung und sogar der eigenen Präsidialadministration (Агентcтво 2022) ist ohne die dem Krieg vorausgehende Personalisierung der Putinschen Herrschaft nicht zu verstehen. Maßgeblich für Russlands regressiven Weg in den Krieg war jedoch der spezifische Kontext der „Ukraine-Frage“. In der Auseinandersetzung mit den politischen und gesellschaftlichen Entwicklungen in der und um die Ukraine radikalisierte und pathologisierte sich Russlands Imperialismus. Zwar war imperiale Rhetroik bereits seit 2012 ein wichtiger Bestandteil des souveränistischen Elitenprojektes. Dem – in der Praxis unerreichbaren – Ziel imperialer Verfügung waren aber noch nicht alle anderen außen- und entwicklungspolitischen Ziele untergeordnet.

So stellten insbesondere in der Perspektive der Geheimdienstelite um Putin die Massendemonstrationen in der Ukraine seit dem Winter 2013, die im sog. Euromaidan und dem Sturz Wiktor Janukowytschs mündeten, eine Wiederholung der Vorgänge in Russland vom Winter 2011/12 und auch des sog. Arabischen Frühlings dar, der bereits Gaddafi zum Verhängnis wurde (Gudkov 2015). Die Ablehnung einer Integration in die von Russland angestrebte Eurasische Wirtschaftsunion (EAWU) durch die ukrainischen Protestierenden war ein massiver Rückschlag für die in der Sowjetunion sozialisierte Entscheidungselite um Putin, deren Denken von einer selbstverständlichen Verfügungsgewalt über die „Blockzugehörigkeit“ post-sowjetischer Staaten geprägt ist. Die russländische Propaganda und Putin selbst projizierten nun die externe Dimension des sich bereits formierten Souveränismus – die westliche Bedrohung für Russlands Souveränität – explizit auf ein klar identifiziertes Ensemble an Feinden: auf dem Maidan aktive ukrainische „Nationalisten“ und der diese unterstützende Westen, allen voran die USA und die EU (Pain 2016, S. 58). Bereits im September 2014 hatte Putin gegenüber dem damaligen EU-Kommissionspräsidenten José Manuel Barroso verlauten lassen, Kyijw könne in wenigen Tagen militärisch eingenommen werden (Harding 2014). Derlei Einschätzungen, die sich im Februar 2022 als fehlgeleitet herausstellen sollten, resulteren aus der habituell begründbaren Unfähigkeit der russländischen Elite, eine eigene Akteurhaftigkeit der Ukraine jenseits des Topois der vom Westen gesteuerten ukrainischen Nationalisten und Faschisten anzuerkennen (Schlichte 2022, S. 423–425). Weder der Euromaidan 2013/14 noch die Wahl Poroschenkos 2015 noch der erdrutschartige und durch saubere demokratische Wahlen herbeigeführte Sieg Wolodymyr Selenskyjs 2019 sind in dieser Perspektive Ausdruck ukrainischer Staatlichkeit oder Gesellschaftlichkeit, sondern lediglich eine Abweichung von der von Putin imaginierten imperialen Einheit Russlands (Путин 2021).

Die Annexion der Krim durch Russland im März 2014 und die anschließende militärische Unterstützung pro-russischer Separatisten im Donbass waren somit, nach der weltwirtschaftlich induzierten Erschöpfung der russländischen Rentenökonommie 2008, den Winterprotesten 2011/12 und „Medwedjews Fehler“ in der Libyen-Frage weitere transformative Ereignisse (Sewell Jr. 2005, S. 98), die das regressive Putinsche Projekt in eine langfristige militärische Verursachungskette überführten, die im Februar 2022 kulminierte. Die politische Ökonomie der Gefahrenproduktion (Кордонcкий 2007), in der die interne Ressourcenallokation des Staates von der wahrgenommenen Notwendigkeit der Bekämpfung immer neuer Bedrohungen abhängt, sorgt dafür, dass die russländische Staatselite sowohl auf explizite ukrainische (militärischer und gesellschaftlicher Widerstand) und westliche (Sanktionen) Entwicklungen mit entsprechenden Gegenmaßnahmen reagiert.

Hierdurch reproduziert sich das souveränistische Projekt nicht nur permanent ideologisch, es radikalisiert sich auch. Davon zeugen mehrere seit 2020 publizierte historisch-philosophische Traktate Putins. Am deutlichsten wurde dies in einem langen Artikel „Zur historischen Einheit der Russen und Ukrainer“ (Путин 2021), in dem Putin die ukrainische Eigenstaatlichkeit als künstliche Erfindung darstellte. Der Text folgte auf einen bereits 2020 veröffentlichten Artikel im amerikanischen *National Interest*, in der Putin seine russisch-imperiale Sicht auf die Geschichte des Zweiten Weltkriegs darlegte (Putin 2020). Die Veröffentlichung beider Artikel ging über das Ritual üblicher staatsmännischer Gedenktexte hinaus. Beide Texte sind Indizien für die geschichtspolitische Verengung im Denken Putins und seine „Obsession mit der Vergangenheit“ (Zygar 2022) im Vorfeld des Überfalls auf die Ukraine. Inwiefern Putins radikale Selbstisolierung während der COVID-19-Pandemie sowie seine revisionistische Geschichtslektüre eine zusätzliche Rolle gespielt haben, werden mit etwas Glück zukünftige Generationen von HistorikerInnen rekonstruieren können.

Mehr noch als die ideologische Radikalisierung von Putin und der ihn umgebenden Elite seit 2012 gehört zu den Bedingungen der Möglichkeit imperialistischer Kriegsführung die extreme Regimepersonalisierung, die inhärenter Teil der vertikalen Regression seit 2012 ist (Burkhardt 2022, S. 35). Die Verfassungsreform von 2020, die Putin ermöglicht bis 2036 – seinem 84. Lebensjahr – zu regieren, war lediglich die letzte formale Kodifizierung dieses Trends. Informell setzte die Personalisierung bereits eher ein, indem zentrale Posten in Politik, Wirtschaft, Verwaltung und Kultur mit Putins Freunden und Vertrauten aus der gemeinsamen St. Petersburger Zeit in den 1990er-Jahren oder deren Familienmitgliedern besetzt wurden. Durch teilweise Verheiratungen bilden diese Gruppierungen mittlerweile ein neo-feudales und – imperialismustheoretisch relevant – eher präkapitalistisches Herrschaftsnetzwerk (Shlapentokh und Woods 2007).

Die Entscheidung zur Invasion der Ukraine fiel folglich isoliert von der Gesellschaft, der Bürokratie, ja sogar der Armee. Nicht einmal die eigenen MinisterInnen waren informiert und wurden *ex post* öffentlich genötigt, die Entscheidung im Sinne einer Kollektivverantwortung mitzutragen (Президент Роccии 2022a). Auch die Umsetzung des Krieges folgt personalistischen Mustern. Schattenmobilisierungen sorgen für Nachschub von Soldaten an der Gesellschaft vorbei, feudal organisierte und Putin persönlich unterstellte paramilitärische Truppen, wie das private Militärunternehmen Wagner, die 2016 geschaffene Nationalgarde oder die vom tschetschenischen Stammesführer Ramsan Kadyrow geführten Eliteeinheiten dienen als dem obersten Feldherrn direkt unterstellte Militärarme.

Gefangen in der situativen Logik des Krieges und einer radikalen Umdeutung des nationalen Interesses in eine absolute Souveränität der Herrschaftselite scheint für Putins Staatsklasse das Schicksal Russlands mittlerweile unmittelbar mit der *internen* Dynamik ukrainischer Staatlichkeit verbunden zu sein. Außenminister Lawrow zufolge werde Russland so viel Territorium annektieren und der Krieg so lange dauern, wie es die militärische Gegenwehr der Ukraine gebiete (РИА Новоcти 2022). Führende russländische PolitikerInnen nennen wöchentlich andere Kriegsziele, die von einem Verhandlungsfrieden (Kreml-Sprecher Peskow) über die Entnazifizierung der Ukraine (Staatsduma-Sprecher Wolodin) bis zur Wiederherstellung der Sowjetunion (Verteidigungsminister Schoigu) reichen. Die internen Dynamiken des russländischen Regimes legen nahe, dass derlei Äußerungen in Unkenntnis der ursprünglichen Kriegsziele Putins getätigt wurden, dessen abstrakte Endziele der Zerschlagung ukrainischer Staatlichkeit und politischen Kontrolle über Kyjiw sich als vorerst unrealisierbar erwiesen haben. Der Zusammenhang zwischen vertikaler Regression der russländischen Herrschaft – extreme Personalisierung, De-Institutionalisierung, ökonomische Primitivisierung sowie ideologische Prä-Modernisierung – und horizontaler Regression der Außenpolitik hat damit seinen vorzeitigen post-sowjetischen Höhepunkt erreicht.

## Die Historizität imperialistischer Außenpolitiken

Russlands Außenpolitik trägt zweifellos imperialistische Züge. Das Land hat Territorien in der Ukraine besetzt, annektiert und in dies mittlerweile in der eigenen Verfassung kodifiziert. Der imperiale Herrschaftsanspruch äußert sich nicht zuletzt in den Ideologiefragmenten und der propagandistisch verbreiteten Rhetorik, die die Staatselite spätestens seit Putins dritter Amtszeit und der notwendig gewordenen Rekonfiguration von Herrschaft in den Jahren 2011 und 2012 zur Legitimierung ihrer Außenpolitik benutzt. Gleichzeitig befindet sich Russland seit dem Zerfall der Sowjetunion in einer post-imperialen Konstellation. Das Imperiale hat vor allem in Form verschiedener Atavismen innerhalb eines russischen Nationalismus überlebt (Pain 2016).

Wie die Weltbilder einer von Sicherheitsdenken, Souveränismus und einstiger imperialer Größe besessenen Staatselite zu konkreten imperialistische Außenpolitiken werden, entgleitet jedoch dem Zugriff des Strukturalismus etablierter imperialismustheoretischer Perspektiven. Einerseits entspringen sie politökonomisch nicht den in den kritischen Sozialwissenschaften betonten kapitalistischen Sozialstrukturen, sondern einer Krise der russländischen Rentenökonomie (Hoppe 2023). Der atavistische Imperialismus des Kreml läuft spätestens seit 2014 den Interessen des Großkapitals entgegen (Matveev 2021). Auch die Entgrenzung des Imperialismusbegriffes auf eine von den USA dominierte und mit Gewaltmitteln aufrecht erhaltene kapitalistische und liberale Weltordnung führt in die Irre. Zum einen ignoriert diese Sicht analytisch die interne und vertikale Regression der russländischen Herrschaft, die die Rekonfiguration des Putinschen Projektes erst notwendig machte. Zum anderen verhindert sie ein klares moralisches Urteil über eine unilaterale Kriegsentscheidung, die zu unzähligen Kriegsverbrechen, zivilen Opfern und einer Krise globalen Ausmaßes geführt hat.

Der Beitrag hat die These vertreten, dass Russlands regressiver Weg in den Krieg nur als Prozess zu verstehen ist. Wenig plausibel scheint die Annahme, dass die Entsendung einer Invasionsarmee mit 150.000 Soldaten zur Unterjochung des Nachbarstaates bereits in den 2000er Jahren angelegt war. Stattdessen hat der Beitrag vorgeschlagen, analytisch atavistische Weltbilder, diese ermöglichende materielle Strukturen – Schumpeters „unterstützende Momente“ – sowie sich wandelnde historische Kontexte zusammenzudenken. Russlands Imperialismus sollte als Prozess und Projekt in einem „einheitlichen historischen Feld“ (Wolfe 1997, S. 407) begriffen werden. Die historisch sensible Imperialismusforschung, die sich nicht dogmatisch der orthodoxen Kapitalismuskritik unterordnet, stellt hierfür methodologische und konzeptionelle Heuristiken bereit, die entsprechende Historisierungsversuche auch für andere Fälle anleiten können. Hierzu zählt Schumpeters Konzept des imperialistischen Atavismus – ein reliktes Elitendenken, das in eigentümlichem Widerspruch zu den gegenwärtigen Sozialbeziehungen steht, jedoch ob deren Krisenhaftigkeit paradoxerweise reproduziert wird. Auch Kielys Idee des Imperialismus als konkretes Projekt sowie Geyers Hinweis auf das Zusammenspiel von krisenhaften internen Entwicklungen und internationaler Politik stellen wichtige Anknüpfungspunkte dar.

Das Verständnis des russländischen Imperialismus als Prozess und Projekt problematisiert nicht zuletzt das In-Eine-Reihe-Stellen der Außenpolitik Russlands mit dem vermeintlich „normalen“ Verhalten anderer historischer und gegenwärtiger Imperien (Münkler 2005). Damit soll keinesfalls jeglicher Vergleichbarkeit von Imperialismen der Boden entzogen werden. Allerdings scheint es geboten, imperialistische Außenpolitiken im Lichte ihrer historischen Diversität zunächst als strategische Projekte *sui generis* zu begreifen, die von konkreten Eliten vorangetrieben werden und keinem transhistorischen imperialen Strukturzusammenhang folgen (Teschke und Lacher 2007).

Zur Historizität des modernen russländischen Imperialismus gehört dann auch die Widersprüchlichkeit aus im globalen Vergleich bestehender ökonomischer Rückständigkeit und imperialem Anspruch der Staatselite. Dieser Widerspruch scheint den tragischen historischen Prozess der internen Selbstkolonialisierung heute unter völlig anderen Kontextbedingungen fortzuschreiben (Koplatadze 2019; Etkind 2011). Russlands regressiver Weg in einen imperialistischen Angriffskrieg anno 2022 bedeutet eben keinen Wandel zu einer umfassenden – man mag sagen, weitsichtigen – außenpolitischen Strategie eines Imperiums. Langfristig bedeutet es die Unterminierung der eigenen wirtschaftlichen und politischen Position durch den pathologischen Souveränismus einer personalistisch vermachteten Herrschaftselite. Deren Streben nach Kontrolle über die ukrainische Politik und Teile des post-sowjetischen Raums bleibt ohne Möglichkeit der faktischen Herstellung von Herrschaft. Es ist damit das Gegenteil des von den kritischen Sozialwissenschaften ins Auge gefassten und von den USA dominierten, liberal-kapitalistischen *empire* des 20. Jahrhunderts (Cumings 1993). Russland fehlen jenseits des Militärs die Mittel der Durchsetzung einer imperialen Herrschaft über die Territorien, die seine politische Elite beansprucht.

Russlands Krieg gegen die Ukraine als vorläufiges Ergebnis eines historischen Prozesses – mit all seinen intendierten und unintendierten Konsequenzen (Hill 2018) – und nicht als strukturell vorbestimmte Unausweichlichkeit zu verstehen, lässt ihn politisch umso schwieriger lösbar erscheinen. Weder reicht es, einem Wandel im Denken der russländischen Staatselite das Wort zu reden, das westliche Sanktionsregime zu verschärfen oder die Ukraine militärisch zu unterstützen – so notwendig und geboten diese Maßnahmen dem Autor dieser Zeilen auch scheinen. Hinter dem Willen zum Krieg steht Russlands nunmehr fünfzehnjährige, verhärtete und sich aus internen, externen und kontextuellen Faktoren speisende Regression, für deren Umkehr die friedenspolitischen Rezepte jenseits eines Pazifismus von der Seitenlinie noch zu fehlen scheinen.

## Footnotes

1 Dt.: Plündern, Morden, Rauben nennen sie mit falschem Namen „Imperium“, und wo sie eine Wüste schaffen, nennen sie es Frieden.

2 Чернышова (2022), Übersetzung durch Autor.

3 Russländisch wird im Folgenden für das Wort *rossijski* benutzt, welches sich auf Aspekte bezieht, die mit der Staatlichkeit Russlands in Verbindung stehen. Russisch hingegen wird für das Wort *russki* verwendet und bezeichnet ethnisch Russisches.

4 Zu den sowjetischen Ursprüngen der Bezeichnung „speziell“ siehe Busygina (2022).

5 Der Beitrag leistet keine Auseinandersetzung mit der Frage nach dem kolonialialen Verhältnis zwischen der Ukraine und Russland, die im Lichte des russländischen Krieges neue Aktualität erfahren hat. Siehe für eine Übersicht über die Debatte Velychenko (2002).

6 Etkind (2011, S. 41) vermutet, dass das Ausblenden sowjetischer und post-sowjetischer Imperialität seitens linker Intellektueller möglicherweise darauf zurückzuführen ist, dass die Sowjetunion insbesondere von Akteuren der sog. Dritten Welt als Inspiration und Model für die eigenen Emanzipationsbewegungen wahrgenommen wurde.

7 Das Verständnis des Imperialismus als Atavismus geht auf die imperialismustheoretischen Arbeiten Joseph Schumpeters zurück. Dieser hatte argumentiert, dass Imperialismus als Summe psychischer Dispositionen und sozialer Strukturen zu verstehen sei, die ihren Ursprung nicht in gegenwärtigen, sondern früheren historischen Kontexten haben. Sog. „unterstützende Momente“, vor allem „innerpolitische Interessenlagen“ würden die Reproduktion kriegerischer Dispositionen und imperialistischen Krieg ermöglichender Sozialstrukturen sicherstellen. Siehe insbesondere Schumpeter (1919, S. 49).

8 Siehe für eine Reflexion der Schwierigkeiten, mit denen eine Narration dieser historischen Rekonstruktionen verbunden ist Knöbl (2022).

9 Morozovs (2015) Begriff des subalternen Imperiums zielt ebenfalls auf die Doppelbewegung aus aggressiv-defensiver Haltung nach außen und imperialem Selbstverständnis.

10 In loser Anlehnung an die Freudsche Psychoanalyse kann unter Regression ein oft selbstschädigender Abwehrmechanismus verstanden werden, der aus unverarbeiteten internen Konflikten in der Vergangenheit resultiert. Siehe Freud (1964).
